# 2D map projections for visualization and quantitative analysis of 3D fluorescence micrographs

**DOI:** 10.1038/srep12457

**Published:** 2015-07-24

**Authors:** G. Hernán Sendra, Christian H. Hoerth, Christian Wunder, Holger Lorenz

**Affiliations:** 1 Center of Molecular Biology, University of Heidelberg (ZMBH), Heidelberg, Germany; 2 Institut Curie -Centre de Recherche, Endocytic Trafficking and Therapeutic Delivery group, Paris, France; 3 CNRS UMR3666, INSERM U1143, France

## Abstract

We introduce Map3-2D, a freely available software to accurately project up to five-dimensional (5D) fluorescence microscopy image data onto full-content 2D maps. Similar to the Earth’s projection onto cartographic maps, Map3-2D unfolds surface information from a stack of images onto a single, structurally connected map. We demonstrate its applicability for visualization and quantitative analyses of spherical and uneven surfaces in fixed and dynamic live samples by using mammalian and yeast cells, and giant unilamellar vesicles. Map3-2D software is available at http://www.zmbh.uni-heidelberg.de//Central_Services/Imaging_Facility/Map3-2D.html.

In order to perform spatial imaging in light microscopy three dimensions need to be considered, which are the lateral (X and Y) and axial (Z) dimensions^1^. For displaying such 3D image data (e.g. from confocal Z-series), stacks of 2D images that show one image at a time, or 2D projections (of the maximum, sum, mean intensities, or texture-based volume renderings, surfaces or orthoslices) are usually presented. A third option is the use of image galleries, which put a collection of images next to each other in form of a single rather disconnected montage. While all of the abovementioned presentation methods have their advantages, they all fail to display the full 3D surface information content as a single, structurally connected 2D image (Supplementary Fig. 1). As a consequence, there is always some image content that remains absent, lost or altered with any of these display options. Since this is presenting a particular challenge for qualitative and quantitative analyses of structurally connected, multiple images-spanning signals, we sought to overcome such limitations. We developed a new software application in MATLAB (MathWorks), called ‘Map3-2D’, to visualize surface data from 3D fluorescence microscopy. We implemented map projection algorithms used in cartography to unfold spatial fluorescence image data onto a single structurally connected map (Fig. 1). A similar approach has recently been published in order to reduce data rate in storage-demanding selective-plane illumination microscopy of Zebrafish embryos^2^. Here, we extent the application range of map projections to quantitative analyses of commonly used (and often smaller-scale) translucent biological samples like cells and subcellular components. Our goal was to develop a software that fulfills the following criteria for map projections: (a) the visualization of 3D fluorescence microscopy data not only for spherical and ellipsoidal surfaces, but also for relatively uneven surfaces containing indentations and protrusions, (b) the option to select regions of interest on the map for further quantification and analysis by cross-referencing with the original 3D image data, (c) the implementation of the time (t) dimension, to evaluate dynamic 3D processes, and (d) the presentation of a ready-to-use software as a platform-independent stand-alone application, free of charge, to everybody who is interested, without the need of a programming background.

We tested the Map3-2D software by applying image sets from a wide range of biological samples with different kinds of scientifically interesting surfaces, as well as artificially created image stacks (Fig. 2). In short, all biological specimens were fluorescently labeled, and series of micrographs were acquired along the axial dimension (i.e. Z-stacks) to get the full surface content of the translucent sample. It is worth mentioning that Map3-2D does not require special 3D image acquisition procedures, which means that already existing image stacks can also be analyzed. As file format, Map3-2D software relies on TIFF (tagged image file format) images to perform the unfolding of the surface information from the image series into an interconnected 2D map.

For a first evaluation of map projections we decided to use artificially created image stacks with surface objects containing geometrical patterns. As opposed to fluorescence micrographs, artificial images do not suffer from optical limitations like spherical aberrations and diffraction. A precise and detailed presentation on 2D map projections could thus be tested and confirmed with these samples (Fig. 2a and Supplementary Fig. 1f). The whole surface content was fully projected and the geometrical patterns accurately reproduced.

We regarded samples whose surfaces are of particular scientific interest to be especially suitable for 2D map projections. As an example we chose giant unilamellar vesicles (GUVs), which are commonly used to visualize phase separation and lipid rafts^3^. We took confocal images of a GUV consisting of two fluorescently labeled lipids and created map projections with Map3-2D. Again, the complete surface information of the GUV could be displayed as a structurally connected image (Fig. 2b). Of note, the user can freely choose how the resulting 2D map will be presented. Map3-2D offers various projections dependent on whether the surface information content is unfolded along the X, Y or Z- axis, and whether the map images are displayed pixel-interpolated or not (Supplementary Fig. 2 and 3). Map3-2D also currently offers nine different map projection algorithms^4^ (Equirectangular, Mercator, Braun, Miller, Lambert-cylindrical, sinusoidal, Mollweide, Wagner VI and Bonne) for optimal presentations of diverse data sets (Supplementary Fig. 4 and Supplementary Table 1 for a summary of the different projections’ properties).

As a biological specimen we chose *S. cerevisiae* bud scars. These are chitin deposits on the cell wall resulting from cell divisions of yeast cells^5^. Unlike the previous examples, the bud scar signals were not fully covering the whole surface. Instead, the scars are distinct ring-like structures across the cell wall with signal-free areas between them. Since it was hardly possible to display each individual scar and its distribution in a single quantifiable image by any conventional display method, we tested whether Map3-2D software could provide such information in a single 2D map. We acquired Z-stacks of fluorescently labeled wheat germ agglutinin-treated yeast cells on a widefield microscope and subjected the images to the software. The resulting 2D map projection clearly and accurately showed both the total number and the distribution of all bud scars in one interconnected image (Fig. 2c).

Since the majority of biological samples do not present perfectly smooth surface shapes, we looked for solutions to display membrane undulation on a 2D map. The generation of map projections relies on reference ellipsoids to perform the unfolding of the 3D surface data onto 2D maps (further explained in Supplementary methods). In addition to intensity value projections (as shown in Fig. 2a–c), this also offers the possibility to project the surface’s height distribution by using the reference ellipsoid as an elevation reference (i.e. baseline). To put it in a cartographical context, the reference ellipsoid can be compared to the geographical mean sea level, and the object’s surface represents the (positive, zero or negative) height above, on or below it. Similar to hypsometric tints on cartographic maps the height differences between a surface and its reference ellipsoid can be assessed and color-coded to indicate deviation from the baseline (protrusions and indentations). The color-coding can be used to describe the shape of a surface and the change of shape over time with respect to a reference ellipsoid. As an example for non-ellipsoid uneven surfaces we chose the outer nuclear membrane of a mammalian cell. Since it is contiguous with the endoplasmic reticulum (ER), we took confocal Z-stacks of NRK cells expressing the ER transmembrane protein CD3δ-GFP^6^, extracted the nuclear surface signal by image processing and subjected the image series to the Map3-2D software (Fig. 2d). The resulting projection showed the corresponding height map of the nuclear surface, color-coded to display the areas of protrusion (positive elevation) and indentation (negative elevation). Changes of the nuclear shape over time could thus be examined and evaluated by analyzing 2D map projections from image stacks taken at different time points (Supplementary Videos 1–3).

To further explore the usability of map projections for dynamic analyses of biological samples we subjected 4D image stacks (i.e. 3D imaging over time) to the Map3-2D software. The only limiting factor with such experiments is the additional time needed to acquire the whole image stack instead of a single layer. However, since many diffusion rates of proteins (including GFP chimeras) can be sufficiently analyzed within the higher milliseconds to lower seconds range^7^,^8^, most motorized widefield setups and many confocal microscopes (for example spinning disk microscopes, Leica’s resonant scanner, Zeiss LIVE) allow for such high data acquisition rates. The advantage of image stacks over single frames in combination with the Map3-2D unfolding is that the whole surface data content can be visualized and inspected on a single map projection image. This way, no biological signal or structure of interest will ever be lost and therefor omitted from the image analysis, simply because it was not located on the single frame acquired, as done in conventional 2D time-lapse imaging experiments. Also, any intensity value-based image analysis will become more complete, and hence more accurate with the Map3-2D software, as the evaluation is based on the entirety of the surface data instead of solely the sub-content from a single layer. Since Map3-2D’s map projections cross-reference with the original data and vice versa, regions of interest (ROIs) can conveniently be selected on the map projection image. Thereupon images get precisely analyzed from the corresponding area on the raw data image stack (Fig. 1).

We tested the Map3-2D software to analyze protein dynamics from photoactivation^9^ (Fig. 3 and Supplementary Fig. 5 and Supplementary Video 4), fluorescence loss in photobleaching (FLIP) (Supplementary Video 5) and fluorescence recovery after photobleaching (FRAP)^7^ (Supplementary Fig. 6) experiments in mammalian and yeast cells. For photoactivation, the ER signal of CD3δ-PAmCherry^6^ was locally switched to red fluorescence^10^ on the back-lateral side of N2a cells. Its diffusion across the ER was recorded over time by confocal microscopy using a resonant scanner, and the 4D image stacks were subjected to Map3-2D software (Fig. 3a). Here, the whole CD3δ-PAmCherry ER signal was considered as being the heterogeneous surface to be unfolded around the nucleus (Fig. 3b). We measured the diffusion of CD3δ-PAmCherry across the full mid-horizontal surface section by plot profiling on the map projections at different time points (Fig. 3c). This kind of 3D analysis is difficult to accomplish by using conventional display methods. However, by using map projections and Map3-2D this becomes a straightforward application. We also measured the mean intensities for different ROIs of the ER over time (Fig. 3a,d). Since the map projections provided structurally connected images of the ER surface at each time point, ROIs could easily be selected and analyzed. Compared to conventional intensity measurements of single Z-layer images, the analysis from map projections showed a much smoother intensity distribution over time (Fig. 3a,d, compare curves for whole surface and single Z-layer). As the surface measurement is based on the complete data content, variations between individual Z-layers did not affect the overall result of the analysis (Supplementary Fig. 5).

We have demonstrated the benefits of map projections for a wide range of biological specimens. The key advantage of our software is that complex surface data from 3D micrographs can be displayed as a single structurally connected projection image. By cross-referencing between this image and the original image stack, precise quantitative analyses of intensities and shapes can be easily and intuitively executed.

## Methods

### Image processing and analysis

The self-written macros Sphere_Builder and Ellipsoid_Builder were used to create all artificial image stacks. The macros can be downloaded freely at http://imagej.nih.gov/ij/macros/. Texture-based volume renderings of image stacks were generated with the ImageJ 3D viewer^11^. General image processing and final image preparations for publication were performed by using Photoshop (Adobe) and ImageJ/FIJI^12^,^13^.

### Preparation of giant unilamellar vesicles (GUVs)

GUVs were prepared as described previously^14^. In short, lipid mixtures (Avanti Polar Lipids, Alabama, USA) were prepared at 5 mg/ml, uniformly mixed, spread evenly onto indium-tin oxide covered glass slides (Delta Technologies), dried under vacuum for at least 2 h, placed into a custom-made Teflon chamber, separated by a 2 mm thin spacer, and filled with a sucrose solution (600 mM). An electric AC-field (1.1 V, 10 Hz) was applied for 2 h at 60 °C following the previously described GUV electroformation protocol^15^. Lipid mixture: DPPC (53 mol%), DOPC (27 mol%), cholesterol (20 mol%), NBD-DPhPE (0.5 mol%) and lissamine-rhodamine-PE (0.1 mol%). Liposomes were harvested after cooling to room temperature. A 200 μl observation chamber (Lab-Tek 8-well chambered #1.0 borosilicate or ibidi 8-well μslide) was coated using 2 mg/ml BSA to avoid distortion of GUVs upon contact with the chamber. Sucrose-containing GUVs were mixed with a 600 mM glucose buffer in order to get a difference in density, which allows imaging of stable and close to the focal plane localized GUVs. The buffer added to GUVs matched the osmolarity of the GUV solution.

### Labeling of *S. cerevisiae* bud scars

Yeast cells were grown overnight, harvested by centrifugation and resuspended in PBS. 500 μl Aliquots of 10^7^ cells were washed twice in PBS and labeled in 500 μl FITC-conjugated lectin from *Tritium vulgaris* (Sigma-Aldrich) in PBS at a concentration of 1 mg ml^−1^. Cells were gently agitated at room temperature for 30 min, pelleted by centrifugation and washed three times in PBS. The labeled yeast cells were resuspended in 100 μl PBS and mounted in ProLong Gold (Life Technologies) for imaging.

### DNA constructs

The plasmid pYFP-GL-GPI and the CFP version, pCD3δ-CFP, of the expression vectors pCD3δ-GFP and pCD3δ-PAmCherry have been described previously^6^,^16^.

### Mammalian cell transfection

NRK and N2a cells were grown in Dulbecco’s modified Eagle’s medium supplemented with 10% (v/v) fetal bovine serum at 37 °C in 5% (v/v) CO_2_. Transient transfections were performed using 25 kDa linear polyethylenimine (Polysciences)^17^. 16–24 h post-transfection the cells were subjected to confocal microscopy.

### Microscopy

Confocal imaging of GUVs, yeast and mammalian cells was performed with a resonant scanner-equipped Leica TCS SP5 using a HCX PL APO 63x/1.20 numerical aperture (NA) water objective lens (Leica) and a Zeiss LSM 780 microscope using a 63x/1.40 NA Plan-Apochromat oil objective lens (Zeiss) and pinhole settings between 1 and 3 airy units. Epifluorescence microscopy of yeast bud scars was performed with an Olympus xcellence IX81 microscope system using a 100x/1.45 NA Plan-Apochromat oil objective lens (Olympus).

## Additional Information

**How to cite this article**: Sendra, G. H. *et al.* 2D map projections for visualization and quantitative analysis of 3D fluorescence micrographs. *Sci. Rep.* **5**, 12457; doi: 10.1038/srep12457 (2015).

## Supplementary Material

Supplementary Information

Supplementary Video S1

Supplementary Video S2

Supplementary Video S3

Supplementary Video S4

Supplementary Video S5

## Figures and Tables

**Figure 1:**
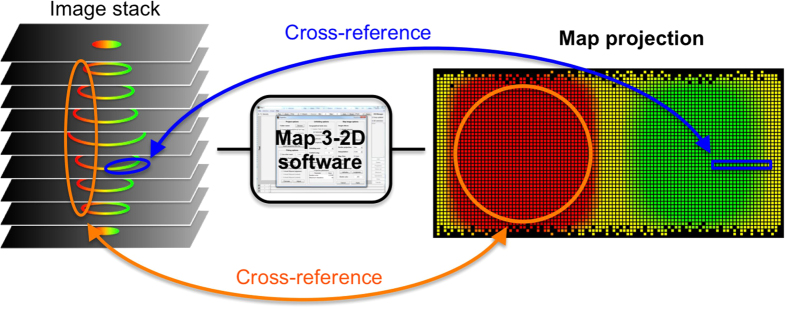
Schematic representation of the of Map3-2D software application. Fluorescence microscopy 3D (up to 5D) image stacks **(left)** serve as input for the Map3-2D software **(middle)**. The whole surface data of the object of interest is projected onto a 2D map image **(right)**. Map3-2D software registers all pixel information of the image stack (intensity, position within the stack, position with respect to the reference ellipsoid). Regions of interest corresponding to single **(blue lines)** or multiple layers **(orange lines)** of the image stack can be selected on the map projection for cross-referencing and quantitative analysis on the original data, and vice versa.

**Figure 2:**
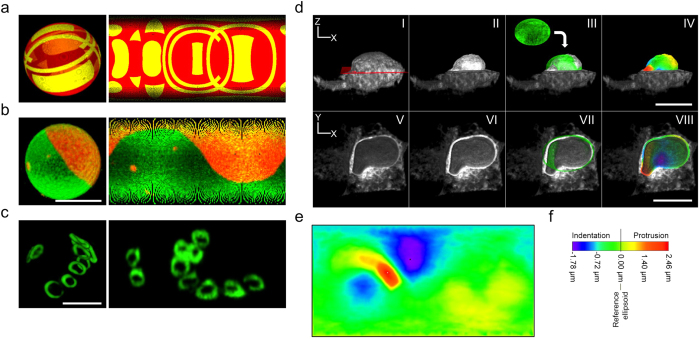
Map projections of surfaces for intensity and shape displays. **(a–c)** Map projections **(right)** from image stacks showing the full surface intensity information of **(a)** an artificially created 3D object, **(b)** a GUV and **(c)** bud scars of a yeast cell. Front views of texture-based volume renderings are shown as reference (left). **(d,e)** Equirectangular map projection as shape descriptor. **(d)** Lateral XZ **(top row)** and axial XY **(bottom row)** views of volume renderings of the CD3δ-GFP ER signal in a NRK cell. All ER signal above a mid vertical section **(red layer shown in d.I)** was removed by image processing to reveal the shape of the nucleus in full **(top row)** or in half **(bottom row)**. A reference ellipsoid **(d.III,d.VII)** was projected onto the nucleus as elevation reference for the map projection. **(e)** Map projection as height map for the whole surface of the nucleus shown in **(d.II-d.VIII)**. Deviations from the reference in form of protrusions and indentations are accurately displayed by color-coding **(f)**. All projections shown are Equirectangular maps, displayed either pixel-interpolated **(c,e)**, or not **(a,b)**. Scale bars, 5 μm **(b,c)**, 10 μm **(d)**.

**Figure 3:**
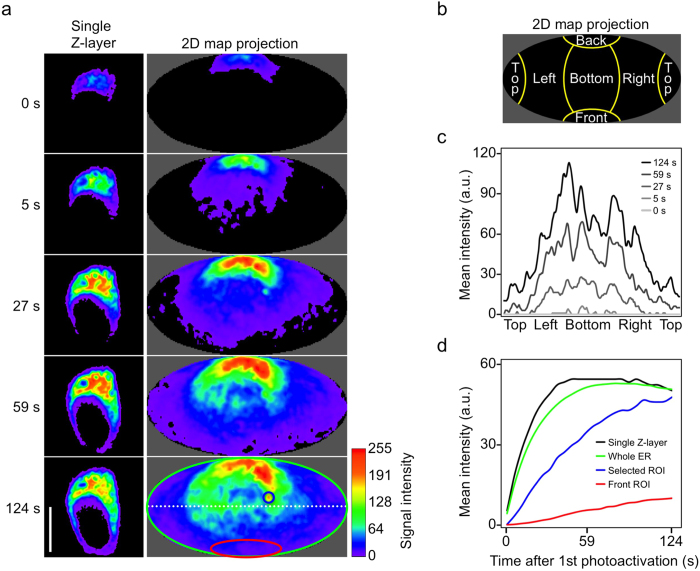
Quantitative analyses with map projections. **(a)** Photoactivation experiment of an N2a cell expressing CD3δ-PAmCherry, displayed as a single Z-layer **(left)** or Mollweide map projection **(right)**. Different time points upon continuous photoactivation are shown, starting at 0 s immediately after the first photoactivation event at the back-lateral side of the cell. The (white dotted) line and (red, green and blue) area selections used for fluorescence signal plot profiling **(c)** and mean intensity area measurements **(d)** are displayed on the map projection at time point 124 s. For orientation, a schematic Mollweide projection **(b)** shows the position of the ER signal with respect to the nucleus as four lateral sides (left, right, back, front) and top and bottom positions (above and below the nucleus). **(c)** Quantitative dynamic analysis of photoactivated CD3δ-PAmCherry diffusion across the full mid-horizontal section of the ER by plot profiling on the map projections shown in **(a)**. **(d)** Quantitative analysis of photoactivated CD3δ-PAmCherry at different ROIs over time. The colors of the mean intensity curves correspond to the colors used for the selections shown in **(a)**. All mean intensities were measured on the original image stacks by cross-referencing the area information from the corresponding maps. For the single Z-layer images, the whole ER area from that single layer was used for measurements. The Mollweide maps are presented as pixel-interpolated, maximum intensity projections. For better intensity discrimination, all images in **(a)** are displayed by using the rainbow smooth lookup table. a.u., arbitrary units. Scale bar, 10 μm.
