# Erratum: 2D map projections for visualization and quantitative analysis of 3D fluorescence micrographs

**DOI:** 10.1038/srep14160

**Published:** 2015-09-21

**Authors:** G. Hernán Sendra, Christian H. Hoerth, Christian Wunder, Holger Lorenz

Scientific Reports 5: Article number: 1245710.1038/srep12457; published online: 07242015; updated: 09212015

The Supplementary Movie legends were omitted from the original version of this Article. The Movie legends appear below:

**Supplementary Video 1**

Dynamic map projection of an artificially created sphere with a moving protrusion. The 3D spherical object (top-left) and the corresponding reference sphere (top-right) are shown as texture-based volume renderings. A Mollweide map projection was used to obtain a color-coded height map (middle), where the reference sphere defines the baseline. The height information was transferred to the original stack of images as an additional channel and rendered in 3D (bottom). Please notice that the moving protrusion is always detectable on the map projection, but not on the texture-based volume renderings. a.u., arbitrary units (voxels).

**Supplementary Video 2**

Dynamic map projection of an artificial sphere with a protrusion reducing its height to become an indentation The 3D spherical object (top-left) and the corresponding reference sphere (top-right) are shown as texture-based volume renderings. A Mollweide map projection was used to obtain a color-coded height map (middle), where the reference sphere defines the baseline. The height information was transferred to the original stack of images as an additional channel and rendered in 3D (bottom). Please notice that the height adopts negative values for indentations below the baseline. a.u., arbitrary units (voxels).

**Supplementary Video 3**

Dynamic map projection of a mammalian cell nucleus. The height information is used to observe surface undulations over time. Image data from a half vertical section of the ER is volume-rendered at the beginning of the video (left). The reference ellipsoid is then estimated (right) and later merged with the ER (left). In order to better visualize the 3D nature of the nucleus, its outer membrane was overlapped to the ER and the reference ellipsoid (left). Finally the color-coded height map is shown on a Mollweide map projection (right), which was also transferred to the original stack of images as an additional channel and 3D volume-rendered over the outer membrane (left).

**Supplementary Video 4**

Photoactivation of the ER protein CD3δ-PAmCherry in an N2a cell, displayed as a Mollweide map projection (left) for the whole ER, or a single Z-layer (right). The photoactivation area is outlined in white. The increase of signal upon continuous photoactivation over time is shown. For better intensity discrimination, the rainbow smooth lookup table was applied.

**Supplementary Video 5**

FLIP experiment of the membrane protein YFP-GL-GPI in an NRK cell, displayed as a Mollweide map projection (left) for the whole membrane, or a single Z-layer (right). The photobleaching area is outlined in white. The loss of signal upon continuous photobleaching over time is shown. For better intensity discrimination, the rainbow smooth lookup table was applied.

