# Supplementary material for: 2D map projections for visualization and quantitative analysis of 3D fluorescence micrographs
Source: Sci Rep. 2015 Jul 24;5:12457. doi: 10.1038/srep12457 (PMC4513544; doi:10.1038/srep12457)
Supplement: Supplementary Information [file srep12457-s1.pdf]

2D map projections for visualization and quantitative analysis of 3D fluorescence micrographs

Supplementary information

G. Hernán Sendra[1], Christian H. Hoerth[1], Christian Wunder[2,3] & Holger Lorenz[1,*]

[1]Center of Molecular Biology, University of Heidelberg (ZMBH), Heidelberg, Germany. [2]Institut Curie -Centre de Recherche, Endocytic Trafficking and Therapeutic Delivery group, Paris, France. [3]CNRS UMR3666, INSERM U1143, France.

*Correspondence: h.lorenz@zmbh.uni-heidelberg.de

Supplementary information

Content

Supplementary Figures ... 2
Supplementary Figure 1: Display modes for 3D image data ... 2
Supplementary Figure 2: Map displays with and without pixel-interpolation ... 5
Supplementary Figure 3: Different unfolding orientations for map projections ... 6
Supplementary Figure 4: Map projection types of Map3-2D software ... 7
Supplementary Figure 5: Comparison between whole surface data and single Z-layer analysis in a photoactivation experiment ... 10
Supplementary Figure 6: Comparison between whole surface data and single Z-layer analysis in a FRAP experiment ... 12
Supplementary Methods ... 14
Image pre-processing ... 14
Surface Mask ... 14
Supplementary Figure 7: Algorithm for the unfolding process after defining the mask ... 15
Unfolding Process ... 15
Reference Ellipsoid ... 16
Supplementary Figure 8 ... 17
Geographical Coordinates ... 17
Map Projection ... 18
Supplementary Table 1: Map3-2D projections, their mathematical expressions and properties ... 20
Map Image ... 21
Supplementary Figure 9: Scheme of the discrete values in a wide surface mask ... 22
Software Features ... 22
Visualization of Multi-dimensional Images ... 22
Supplementary Figure 10: Software toolbar ... 23
Supplementary Figure 11: Software dialog to define the background value ... 23
ROI Manager ... 23
Supplementary Figure 12: ROI Manager related dialogs ... 24
Open/Save Images ... 25
Quantitative Analysis ... 25
Objects with uneven surfaces: protrusions and indentations ... 25
Supplementary References ... 27

Supplementary Figures

Supplementary Figure 1: Display modes for 3D image data

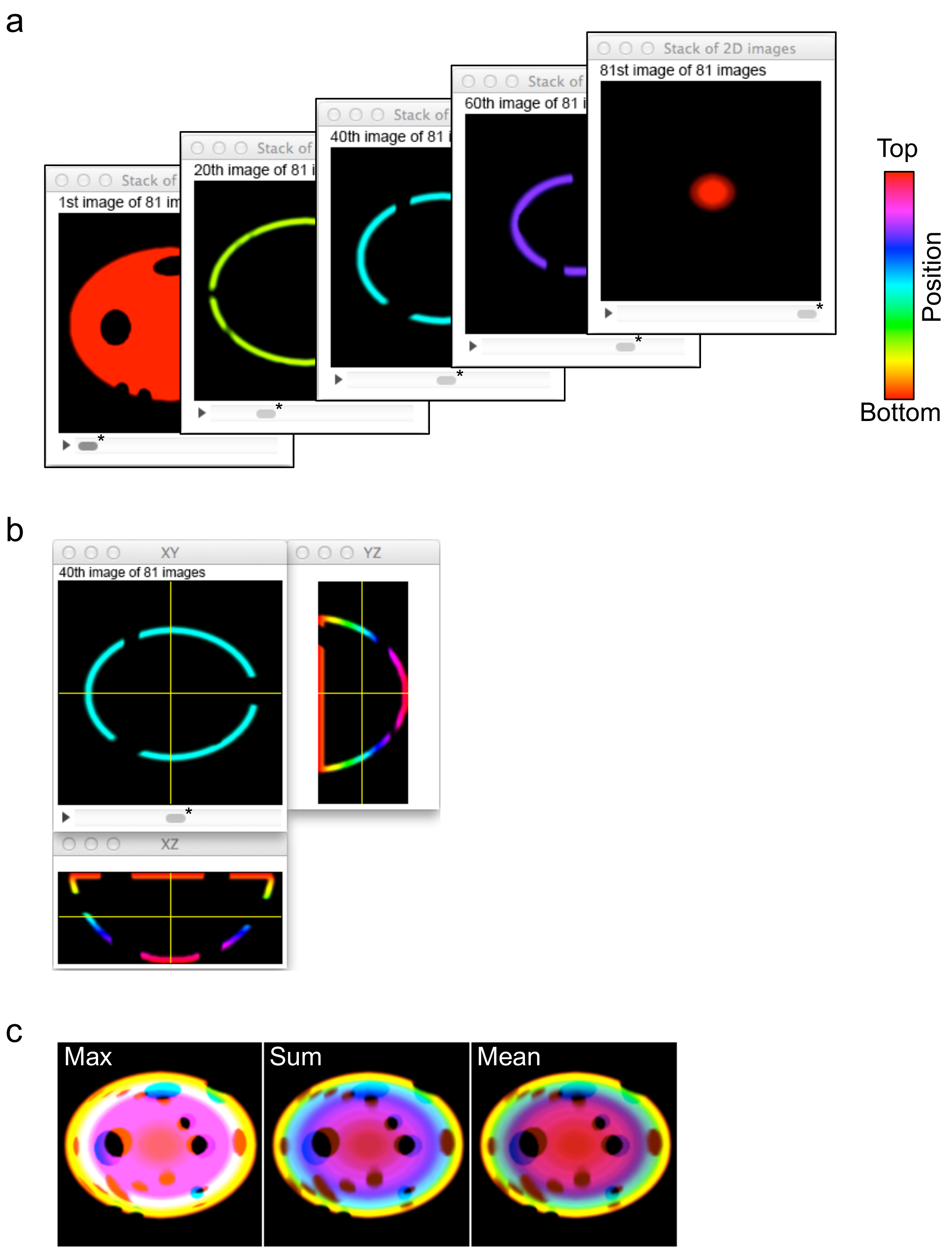

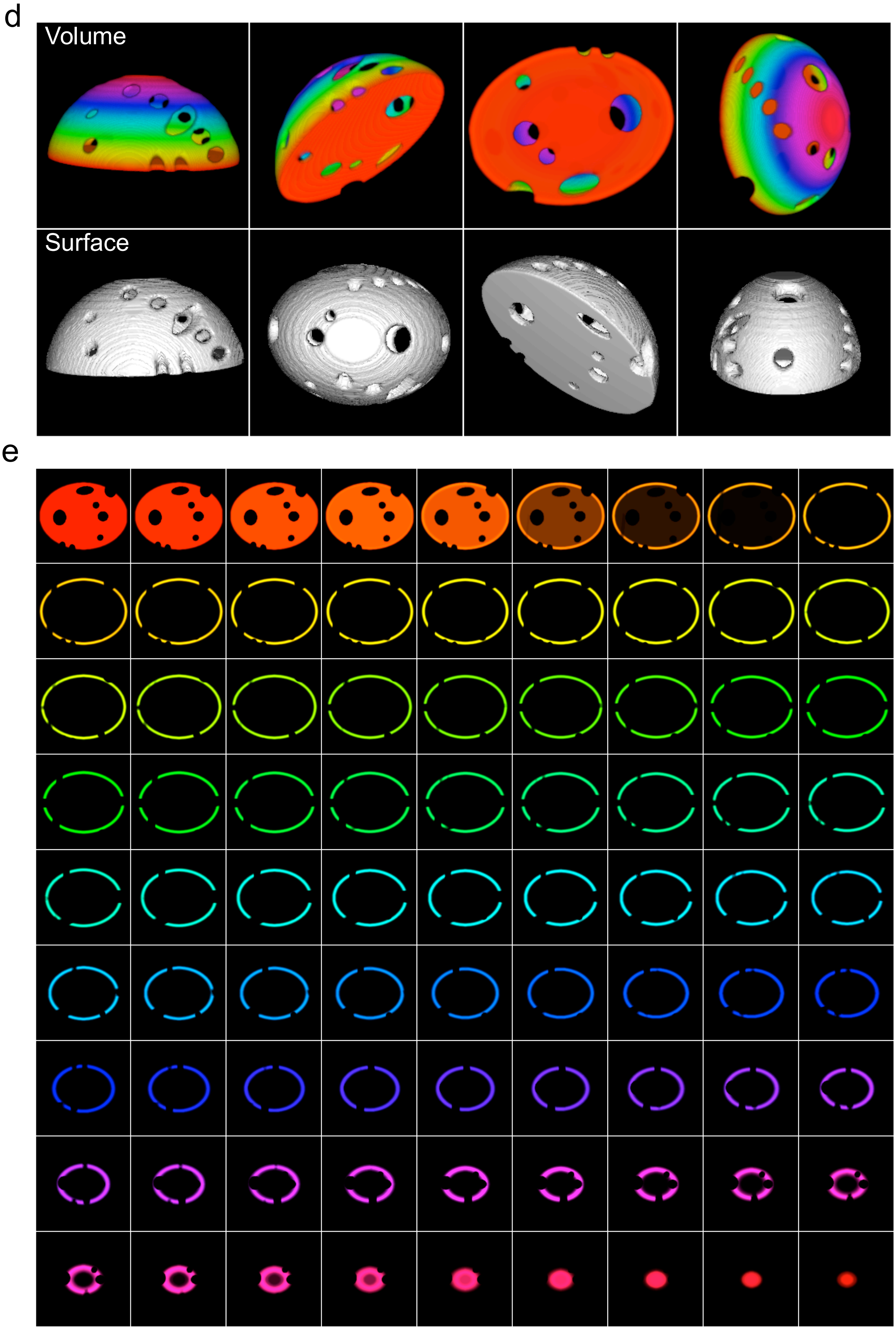
d
Volume
Surface
e

f

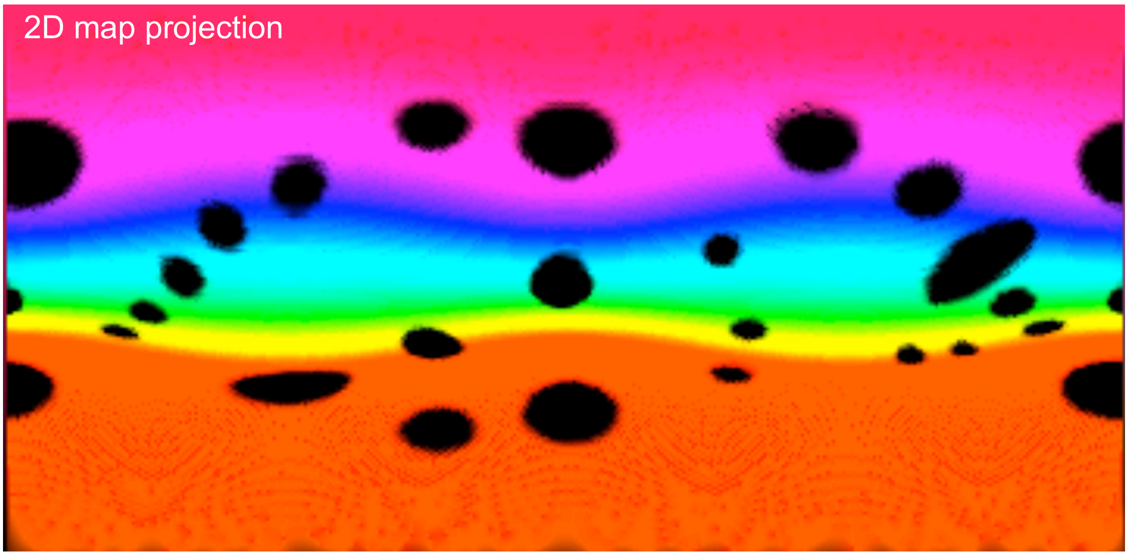

Supplementary Figure 1: Display modes for 3D image data

An artificially created image stack is shown to demonstrate various modes of 3D image displays. The object of the image stack is intended to resemble an adherent cell's surface membrane, with a flat bottom representing the cell's contact with the culture dish. Heterogeneity of the surface is provided by the presence of different-sized holes across the membrane. For orientation, the image stack is color-coded along the axial direction (see reference bar in **(a)**).
Commonly used display modes for 3D fluorescence image stacks are shown in **(a)** - **(e)**. **(a and b)** Dynamic image displays by using a scrollbar(*) to move through an image stack, showing either a single 2D layer **(a)** or three images from different orthogonal views **(b)** at a time. **(c)** Static projections of the maximum (left), sum (middle) or mean (right) intensities. **(d)** Texture-based volume (top row) and surface (bottom row) renderings from four different viewing angles each. **(e)** Image gallery (or montage) with all frames of the image stack put together in columns and rows. **(f)** 2D map as a result of an unfolding event of the image stack by using Map3-2D software. Note that none of the display modes in **(a)** - **(e)** is able to show the full surface content in a single, structurally interconnected image. Only projections, which unfold the surface content onto 2D maps **(f)** generate interrelated images, which allows for an intuitive and straightforward data analysis. Here, a pixel-interpolated Equirectangular map projection, unfolded along the Z-axis is shown (see Supplementary Figures 2-4).

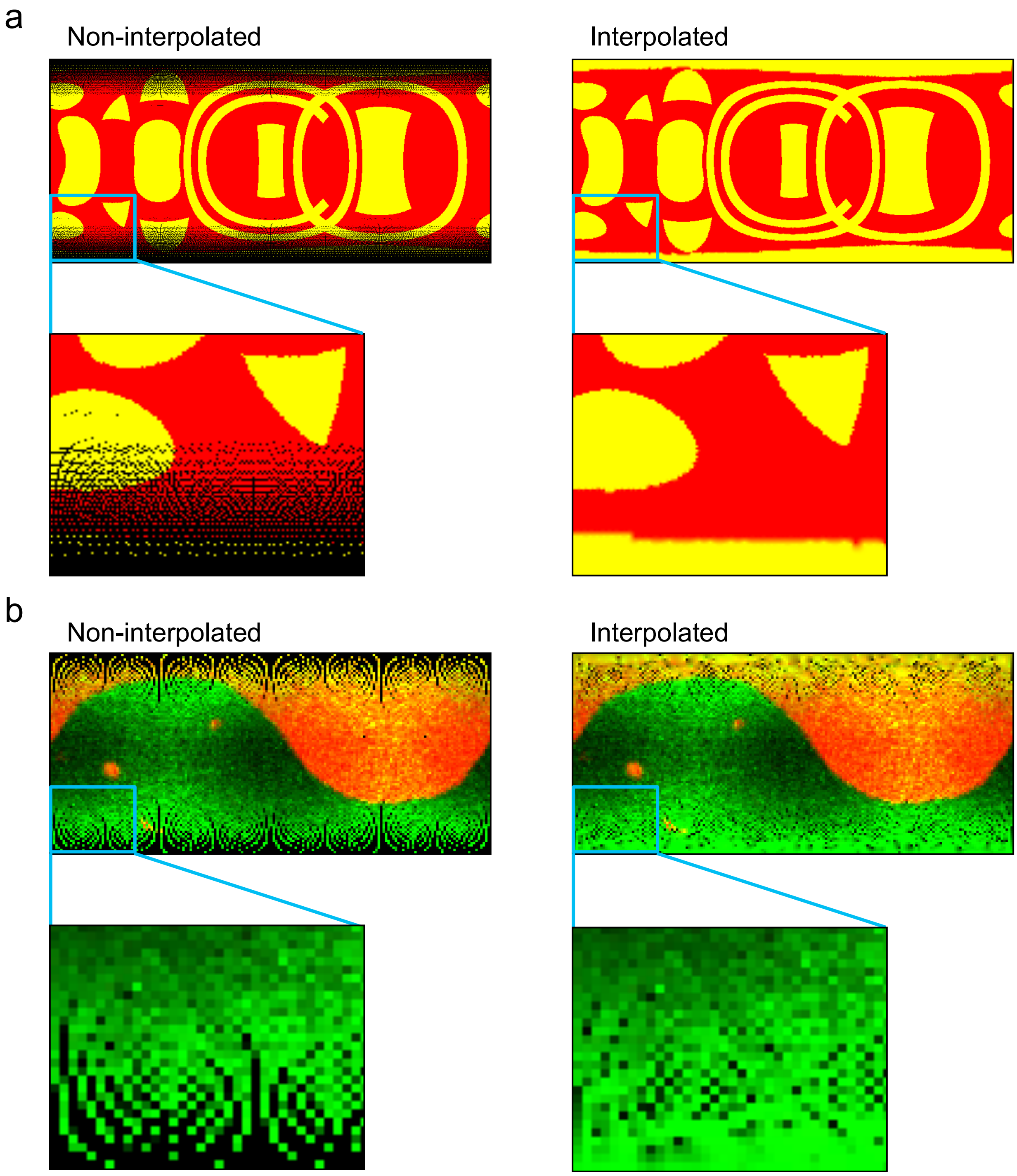

Supplementary Figure 2: Map displays with and without pixel-interpolation

Map projections without **(left)** and with **(right)** pixel-interpolation for **(a)** an artificially created 3D object and **(b)** a GUV (see Figure 2a,b in the main article). For a more detailed presentation of the differences, bottom left regions are magnified and shown beneath.

Su [illegible] **olding**

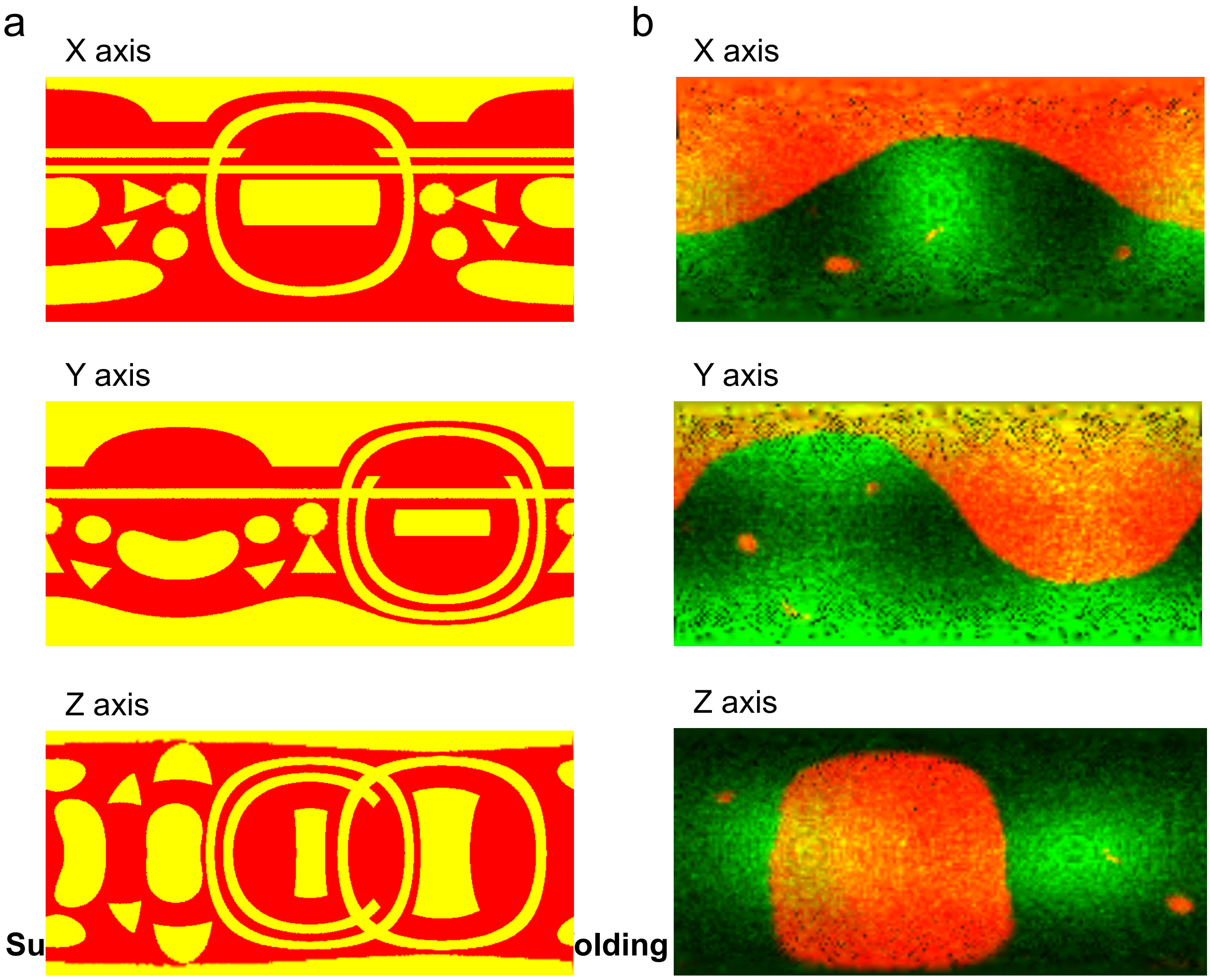

Map projections using different unfolding axes for **(a)** an artificially created 3D object and **(b)** a GUV (see Figure 2a,b in the main article). All map projections are shown as pixel-interpolated images.

Supplementary Figure 4: Map projection types of Map3-2D software

a

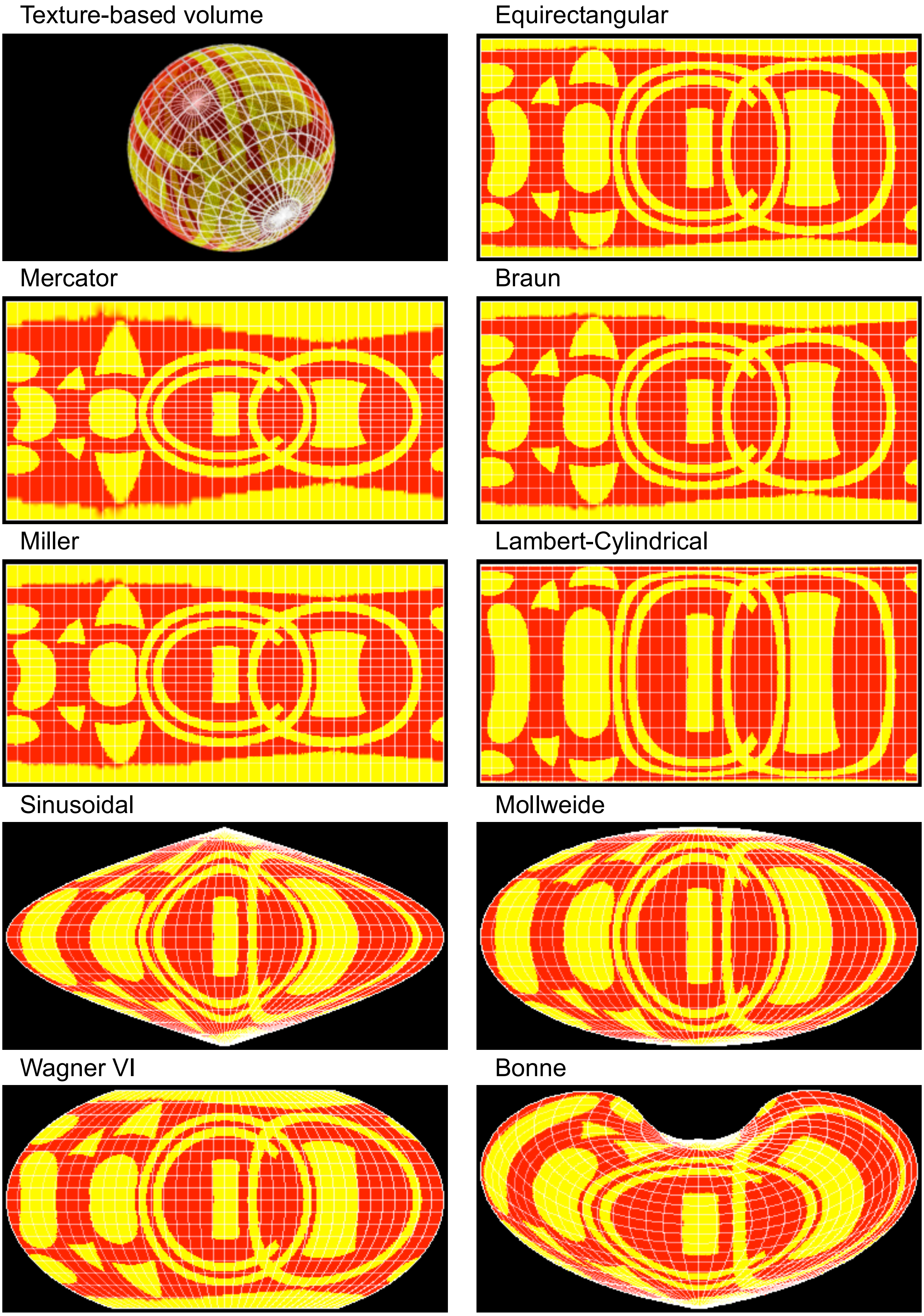

b

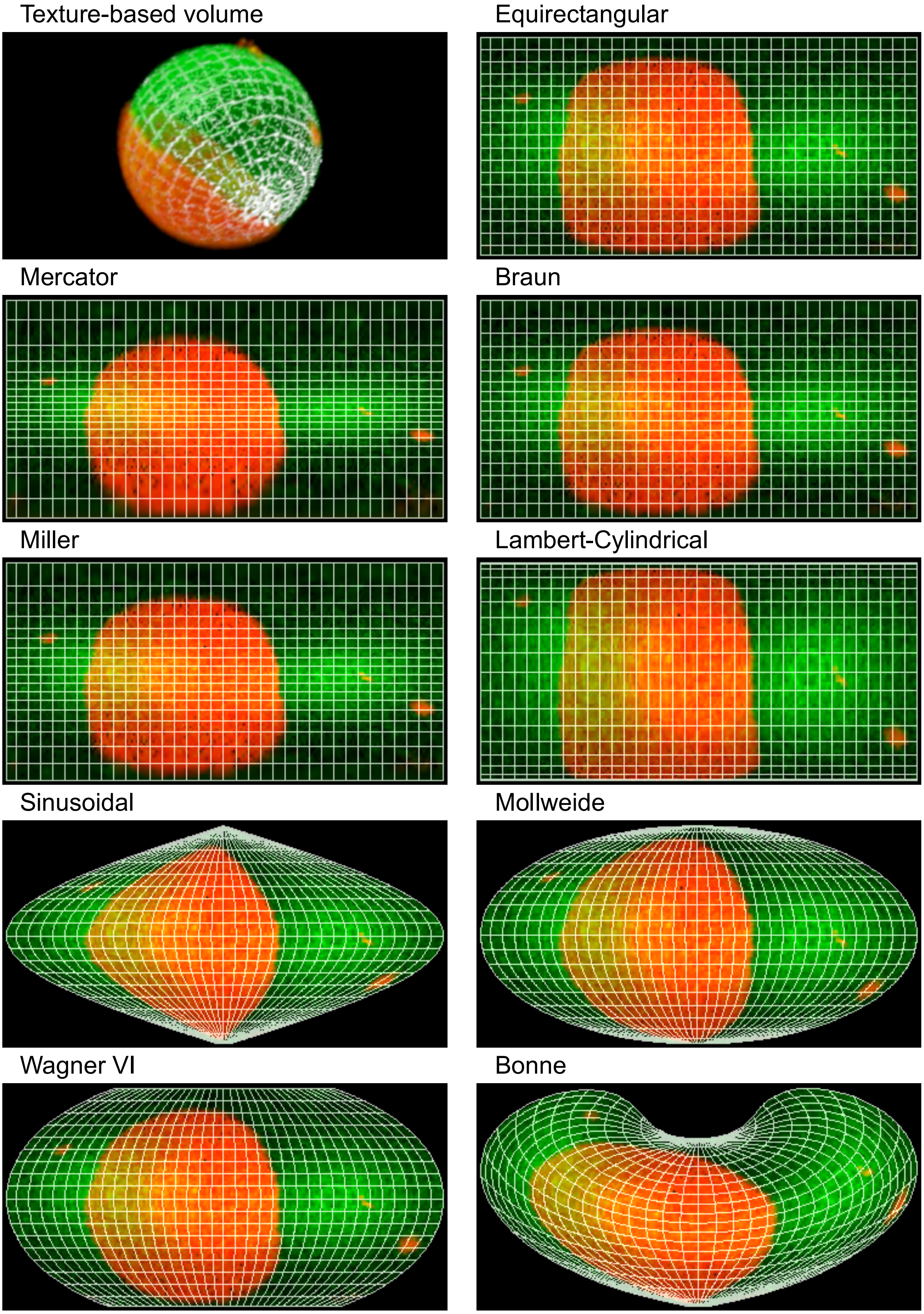
Texture-based volume
Equirectangular
Mercator
Braun
Miller
Lambert-Cylindrical
Sinusoidal
Mollweide
Wagner VI
Bonne

Supplementary Figure 4: Map projection types of Map3-2D software

Different projection types for **(a)** an artificially created image stack representing a sphere object with geometrical patterns and **(b)** a fluorescence image stack of a giant unilamellar vesicle (see Fig. 2a and 2b of the main article). **(left to right and top to bottom)** Equirectangular, Mercator, Braun, Miller, Lambert-Cylindrical, Sinusoidal, Mollweide, Wagner VI and Bonne projections. Latitude and longitude lines (white) are included for better appreciation of the differences between the projections. See Supplementary Table 1 for a comparison of different projection types.

Supplementary Figure 5: Comparison between whole surface data and single Z-layer analysis in a photoactivation experiment

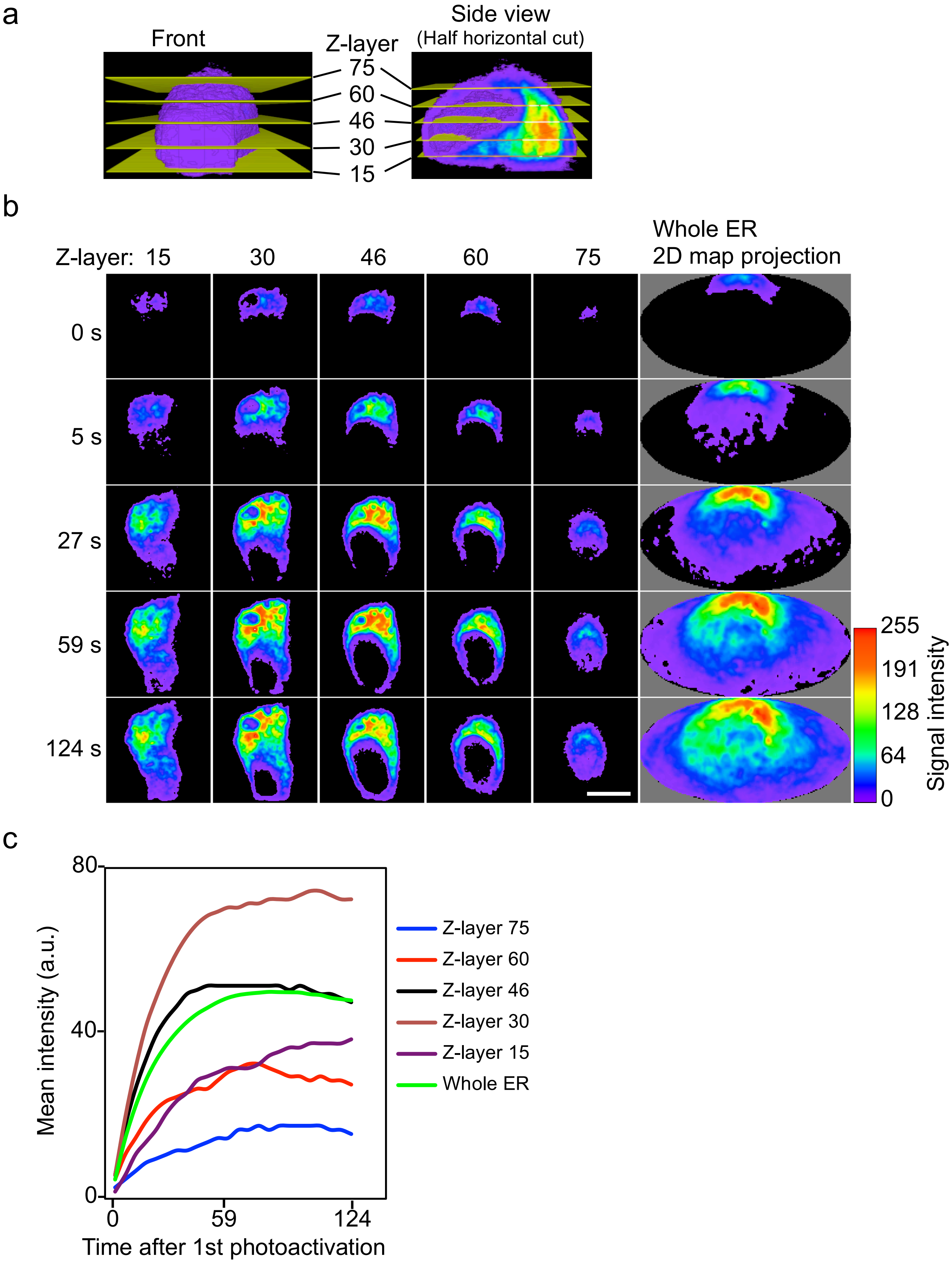

Supplementary Figure 5: Comparison between whole surface data and single Z-layer analysis in a photoactivation experiment

Quantitative analyses of the CD3δ-PAmCherry ER signal in an N2a cell over time (see Figure 3a,d of the main article). **(a)** 3D renderings of the ER show the position of the analyzed Z-layers (yellow slices) along the Z-axis of the cell. **(b)** The CD3δ-PAmCherry mean signal intensity upon continuous photoactivation was measured on 5 individual Z-layer images and on the whole ER surface data from a 2D map projection, and **(c)** plotted against time. For whole surface data analyses, the mean intensities were measured on the original image stacks by cross-referencing the area information from the corresponding map projections. For the individual Z-layer images, the whole ER area from each single layer was used for measurements. Please notice the varying results for the intensity plots of the individual Z-layers, since they are based on limited data sets. The plot from the map projections is based on the complete ER data set, and, hence, more accurate for the whole cell.
The Mollweide maps are presented as pixel-interpolated, maximum intensity projections. For better intensity discrimination, all images are displayed by using the rainbow smooth lookup table. a.u., arbitrary units. Scale bar, 10 µm.

Supplementary Figure 6: Comparison between whole surface data and single Z-layer analysis in a FRAP experiment

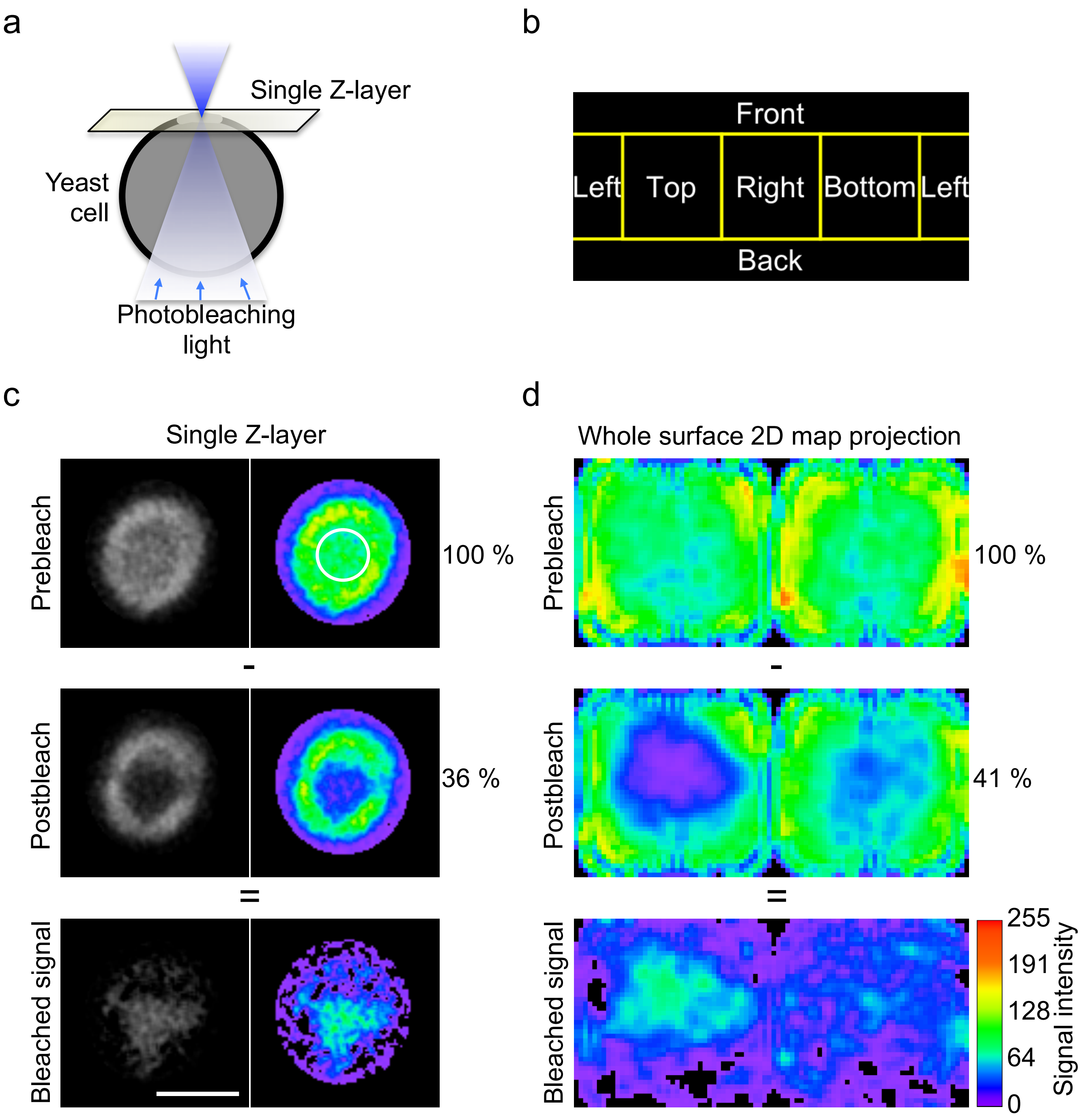

Supplementary Figure 6: Comparison between whole surface data and single Z-layer analysis in a FRAP experiment

Quantitative analyses of the Pma1-GFP membrane signal in a yeast cell before and after photobleaching. **(a)** Schematic representation of the FRAP experiment. The Pma1-GFP expressing yeast cell[1] was placed on an inverted confocal microscope. The fluorescence signal of Pma1-GFP was photobleached at the top of the cell. **(b)** For orientation, a schematic map projection shows the position of the four lateral sides (left, right, back, front) and the top and bottom positions of the yeast cell. (**c**) Images from an individual Z-layer at the top of the yeast cell before ('Prebleach') and after the photobleach ('Postbleach'), shown as grayscale (left) or intensity color-coded (right) images. **(d)** Whole surface map projections from an image stack covering the complete yeast cell before ('Prebleach') and after the photobleach ('Postbleach'), shown as intensity color-coded images. **(c,d, top and middle rows)** Results of the bleach box (white circle) signal intensity measurements for the time points immediately before and after the photobleach are shown next to the images, with the prebleach intensity set to 100 %. **(c,d, bottom rows)** The subtraction of the postbleach images from the prebleach images reveals the Pma1-GFP signal that was bleached ('Bleached signal') on the individual Z-layer **(c)** and on the whole membrane **(d)**.

Notice the significant amount of Pma1-GFP signal that was bleached in the rest of the yeast membrane, i.e. outside the actual bleach box, as revealed by the map projection **(d, bottom row)**. Since quantification from a single Z-layer is based on a limited data set, the quantification will also be limited. FRAP quantification from map projections of the whole membrane is based on the complete data set and will thus be more accurate.

The Equirectangular maps are presented as pixel-interpolated, maximum intensity projections. For better intensity discrimination, all color-coded images are displayed by using the rainbow smooth lookup table. Scale bar, 5 µm.

Supplementary Methods

Image pre-processing

For best results it is advised that the original image stack needs to be background-subtracted and, if applicable, processed further by using filtering techniques and deconvolution. If an image stack (or hyper-stack) involves multiple time frames, signal displacements or rotations might have occurred from frame to frame. In order to correct such signal shifts, registration techniques need to be applied. Here, for 3D registrations of image stacks from time-lapse experiments, a self-written program that uses Matlab's image processing toolbox was employed, and can be downloaded freely at http://www.zmbh.uni-eidelberg.de//Central_Services/Imaging_Facility/Map3-2D.html.

Surface Mask

In order to get a good map projection, one of the more difficult tasks to achieve is the creation of mask images that isolate the surface of the 3D object from non-surface areas. The mask images consist of zero-values from non-surface areas and non-zero values, which represent the surface region of the object of interest (**Supplementary Fig. 7a**). Therefore, the mask is a 3D or 4D stack depending on whether the mask's shape changes over time or not. 4D masks are only necessary when the object displays spatial shape alterations over time, as expected for dynamic live cells.
A preliminary approach to create a simple mask starts with the binarization of the stack by applying a threshold. Morphological 2D or 3D operations like closing or opening can be applied to remove artifacts at the border. The result should be a 3D binary image of the object. If the voxel size is cubic (i.e. same sizes for all three dimensions), a 3D eroded version of the same image can be subtracted to obtain the surface mask. The level of erosion (size of the kernel and/or repetition of the erosion) depends on the desired thickness of the surface of interest. When the voxel is not cubic, more complex operations might be required, like non-cubic morphological filters or combinations of 3D and 2D erosions/dilations.

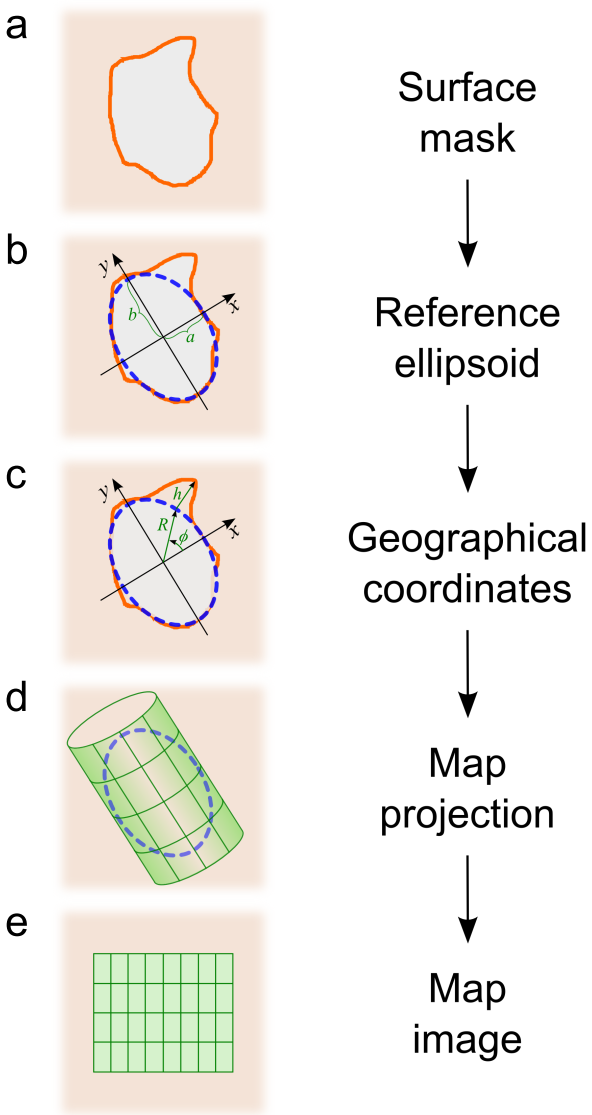

Supplementary Figure 7: Algorithm for the unfolding process after defining the mask

<u>Unfolding Process</u>

Cartography and its terminology have been focused mainly on the Earth with the best example being the *geo* prefix. Here, in order to avoid any confusion with already existing knowledge, the terminology was not altered even though the objects of interest are not planets but microscopic images of biological samples. The process to obtain 2D maps from 3D microscopic surface signals (like cell membranes, nuclei, GUVs, etc.) requires several steps. From the user's point of view, the complexity is relatively high because each step involves parameters that need to be set, and input that needs to be provided.

Sticking to the Earth, its geometry can be approximated by using a (down-scaled) reference ellipsoid (or a sphere in simpler cases), which functions as a mathematical model of the surface without any elevations (protrusions) or depressions (indentations). Similarly, reference ellipsoids can be defined for any kind of 3D surface data, for example from fluorescence image stacks, in order to create 2D maps. The Cartesian coordinates of every point in the surface mask can then be transformed into geographical coordinates with respect to the reference ellipsoid, i.e. latitude, longitude and height. Those coordinates need then to be projected onto a reference ellipsoid, which in turn can be unfolded onto a 2D map. The map itself, which is not a square grid of values, has finally to be converted into an image. How this is done is depicted in **Supplementary Fig. 7** and explained in more detail in the next paragraphs.

Reference Ellipsoid

The unfolding of a 3D surface of interest cannot be done directly on the surface itself. In order to do so, a geometrical reference object is needed (**Supplementary Fig. 7b**). Such an object, either an ellipsoid or in simpler cases a sphere, should ideally match the shape and size of the 3D sample. An ellipsoid centered at the origin and aligned with the coordinate axes can be described by the expression

$$\frac{x^2}{a^2}+\frac{y^2}{b^2}+\frac{z^2}{c^2}=1$$

where *a*, *b*, and *c* are the ellipsoid semi axes or radii. The coordinate system needs to be translated and rotated according to the sample. The Map3-2D software provides an algebraic estimation of the best-fitting sphere, rotationally symmetric (oblate) ellipsoid or tri-axial ellipsoid by solving a system of equations using the original data points (*x,y,z*) and by determining coefficients (A, B, C, D, E, F, G, H, I) of the following implicit equation:

$$Ax^2+By^2+Cz^2+2Dxy+2Eyz+2Fzx+2Gx+2Hy+2Iz-1=0$$

where *A=B=C* and *D=E=F=0* for a sphere; while *A=B* and *D=E=F=0* for an oblate ellipsoid on the z-axis.

However, as this is only an algebraic approximation, it often needs to be manually adjusted in terms of center, rotation, and radii or semi axes length. With Map3-2D the user can visually and interactively move and rotate the ellipsoid as well as change every semi axis (**Supplementary Fig. 8**). It is even possible to have different semi axes length according to the direction, resulting in the so called ‘six-axial ellipsoid’[2] which is especially useful for better approximations of non-symmetric shapes very common in biology, like the egg shape.

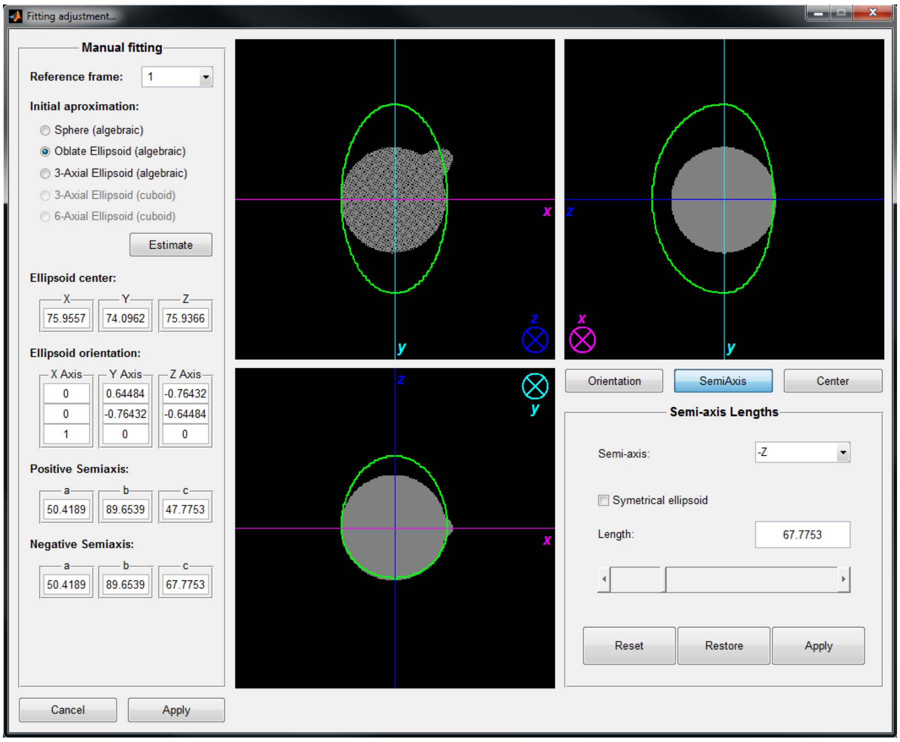

Supplementary Figure 8: Dialog to adjust the reference ellipsoid fitting

Geographical Coordinates

In the next step, every surface position of the sample must be associated with the closest point on the reference ellipsoid. Following an approach used for non-spherical celestial bodies maps[3], the (x, y, z) coordinates of each point are transformed into (latitude, longitude, height) or (ϕ, λ, h) (**Supplementary Fig. 7c**). Latitudes and longitudes are determined through the reference ellipsoid (as in Earth cartography) and the shortest distance between the sample point and the ellipsoid determines the height or elevation. In order to obtain latitude and longitude the user

can decide whether to use the *x*, *y* or *z* dimensions as unfolding axis (analogous to the earth axis). **Supplementary Fig. 3** shows maps using different unfolding axis. The first non-unfolding axis is used as reference to determine the zero longitude. This arbitrary reference longitude or meridian, together with the unfolding axis, can be adjusted to have a desired surface region at the center of the map. The latitudes are calculated using the geocentric approach.

The interpretation of the map latitude and longitude as well as their correspondence to the original images can be assisted by adding a grid of parallel and meridian lines in an additional channel on both the 2D map and the original stack images. The grid is customizable and the user can freely define line position and color (e.g. intensity color-coded).

Map Projection

The geographical coordinates of each position are used to create an intermediate matrix (called 'geographical table' in Map3-2D) whose rows and columns refer to latitudes and longitudes respectively, and each cell's content represents the average height of all the positions whose latitudes and longitudes fall into that cell. The geographical table' points are represented as blue circles on the reference ellipsoid of the scheme illustrated in **Supplementary Fig. 9**. Empty cells are interpolated using the adjacent values. The software automatically estimates the size of this intermediate matrix, however better results can usually be achieved by additional manual readjustments.

The geographical table is used to create the unfolded plane coordinates (*X, Y*) (**Supplementary Fig. 7d**). In maps of the Earth, the information of the heights is usually neglected and the plane coordinates are only functions of the latitude and longitude:

$$X = f_X(\phi, \lambda), \qquad Y = f_Y(\phi, \lambda)$$

Another type of projection, often used in cartography for non-spherical celestial bodies[3,4], also considers the information of the height with the following expressions

$$X = (R + \alpha h) f_X(\phi, \lambda), \qquad Y = (R + \alpha h) f_Y(\phi, \lambda)$$

where h is the height (distance) of each voxel position to the ellipsoid and R is the distance from the ellipsoid's surface point to the ellipsoid axis. An additional height factor α was added to the expression in order to emphasize or deemphasize the influence of the height. The value of R is set by default as the maximum from all the ellipsoid points. The user can decide whether to neglect or consider the heights of the latitudes and longitudes. However, the application of this approach is limited due to the overlap of surface points.

Thanks to an extensive wealth of research in cartography there are many projections with different properties and features[5,6] that can be applied in the Map3-2D software. Some of them differ only in the proportion between height and width of the resulting map. As the actual size (in pixels) of the 2D map is either user-defined or based on the geographical table, those projections lead to the same results. The projections currently available in the Map3-2D are summarized in **Supplementary Table 1**.

Supplementary Fig. 4 shows different projections of the artificially-created 3D sphere and the GUV. If we consider the Equirectangular projection as a reference, the Mercator, Braun and Miller projections are better suited to observe regions close to the poles with higher resolution, despite the deformations associated with the unfolding. However, those projections also decrease the spatial resolution towards the Equator. In contrast, the resolution on the poles is reduced for the Lambert-Cylindrical projection and enlarged towards the Equator. In order to overcome deformations towards the poles, the Sinusoidal and Mollweide projections produce maps where the poles are defined as single points. As a consequence, regions close to the poles undergo less deformation and areas become more comparable across the whole map. In general, Mollweide provides better maps with less deformation than the Sinusoidal projection. The Wagner VI projection provides higher resolution on the poles within a shape similar to Mollweide. The Bonne projection provides good resolution and little deformation over the map except for the regions at the poles.

Projection	$f_X(\phi,\lambda)$	$f_Y(\phi,\lambda)$	Properties
Equirectangular	$X=\lambda\cos\phi_1$<br>ϕ_1: Standard parallel with no scale change	$Y=\phi$	Basic map. Distances along meridians are conserved. Deformations towards the poles
Mercator<br>(Formula corrected only for spheres)	$X=\lambda$	$Y=\ln\left[\tan\left(\frac{\pi}{4}-\frac{\phi}{2}\right)\right]$	Areas get enlarged towards the poles, and comprised towards the Equator
Braun	$X=\lambda$	$Y=\tan\frac{\phi}{2}$	Similar to Mercator, but with less enlargement/compression towards the poles/Equator
Miller	$X=\lambda$	$Y=\frac{5}{4}\ln\left[\tan\left(\frac{\pi}{4}-\frac{2\phi}{5}\right)\right]$	Intermediate between Mercator and Braun
Lambert-Cylindrical	$X=\lambda$	$Y=\sin\phi$	Areas get enlarged towards Equator and compressed towards the poles
Sinusoidal	$X=\lambda\cos\phi$	$Y=\phi$	Poles are single points. Less deformations towards the central latitude and longitude
Mollweide	$X=\frac{2\sqrt{2}}{\pi}\lambda\cos\theta$<br>$2\theta+\sin(2\theta)=\pi\sin\phi$	$Y=\sqrt{2}\sin\theta$	Similar to Sinusoidal, but with less deformations
Wagner VI	$X=\lambda\sqrt{1-3\left(\frac{\phi}{\pi}\right)^2}$	$Y=\phi$	Intermediate between Equirectangular and Mollweide
Bonne	$X=\rho\sin E$<br>$\rho=\cot\phi_1+\phi_1-\phi$<br>$E=\frac{\lambda\cos\phi}{\rho}$<br>$\phi_1$: Standard parallel with no scale change	$Y=\cot\phi_1-\rho\cos E$	Good resolution and little deformation across longitudes, except for the poles

Supplementary Table 1: Map3-2D projections, their mathematical expressions and properties

Map Image

As the (X, Y) coordinates of the projection are real values, they have to be converted into positive integers, which are indices of pixel positions (**Supplementary Fig. 7e**). This process requires usually a stretch or shrink in each dimension since the size of the output map image is either user-defined or automatically defined by the geographical table. Inevitably, stretching or shrinking results in pixels that are either referenced by more than one position, or not referenced at all. Averaging all values that fall into a pixel solves the first case. The second case, however, is more complicated because interpolation is needed. The Map3-2D software solves this problem by allowing the user to decide whether to use interpolation (linear, cubic, natural and nearest), or not **(Supplementary Fig. 2)**.

Due to the discrete nature of the geographical table, more than one position of the surface mask could fall into the same cell of the geographical table. Consequently, these positions will be reduced to a single value. This becomes more apparent when the surface mask is wider than one pixel (**Supplementary Fig. 9**), which is often the case with fluorescence images of biological samples. In order to solve those more-than-one-pixel cases in an appropriate way, Map3-2D software allows the user to apply mean, maximum, minimum or sum intensity projections. On top, the user can always empirically modify the geographical table size, the projection and the mode of interpolation for best map appearances.

The final 2D map is displayed as an extra image. Two additional channels are created, one for the grid and one for heights (or elevation) in relation to the reference ellipsoid. The second one consists of 32 bits floating-point values.

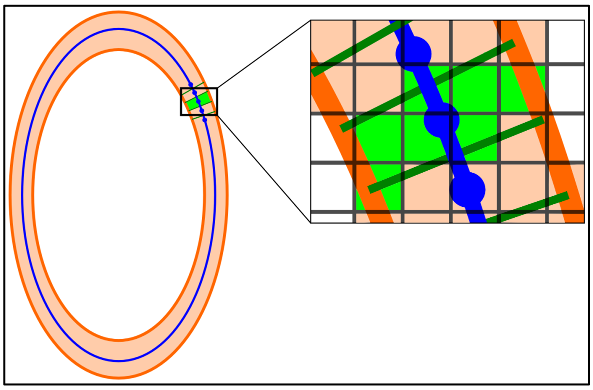

Supplementary Figure 9: Scheme of the discrete values in a wide surface mask The blue line and its blue circles represent the reference ellipsoid and its discrete values according to the geographical table. The green voxels in the zoomed image indicate voxels that fall into the same cell of the geographical table, which will be reduced to a single voxel following the selected border projection method.

Software Features

Besides creating 2D maps, Map3-2D software provides many tools to assist with data analysis, which are described in the next paragraphs.

Visualization of Multi-dimensional Images

In Map3-2D, multidimensional images (or hyper-stacks) are displayed with three scroll bars at the bottom of the image frame to navigate between different channels, z-slices and time frames.

The toolbar (**Supplementary Fig. 10**) contains functionalities to

- zoom in and out
- pan over a zoomed image
- assign different look-up tables or color maps to each channel
- stretch the image
- show only the masked image
- control the display range
- select regions-of-interest within the image

The position of the cursor and the corresponding pixel intensity value are displayed at the left side of the toolbar (**Supplementary Fig. 10**).

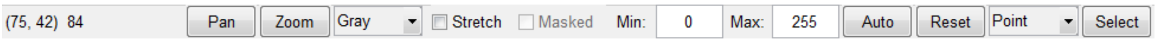

Supplementary Figure 10: Software toolbar
The current pixel position (current image of the stack) and its value are shown on the left.

Pixels outside the mask are considered background, and can be displayed with user-defined values for each channel individually (**Supplementary Fig. 11**). The background pixels are displayed in the output 2D map image when no interpolation is used.

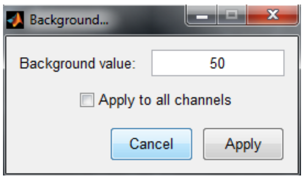

Supplementary Figure 11: Software dialog to define the background value

ROI Manager

Regions-of-interests (ROIs) can be selected on the images and added to the ROI manager, which is displayed on the right side of the graphical interface (**Supplementary Fig. 12a**). There are four main processes that can be done with a ROI:

- *Transfer.* ROIs can be transferred from the 2D map onto the original image stack, and vice versa (**Supplementary Fig. 12b**). As a consequence, a 2D ROI on the map will be converted into a 3D ROI on the original image stack. Precise quantitative area measurements on the original images are therefore feasible (see Quantitative Analysis below).
- *Measurements.* The data contained in each ROI can be analyzed by measuring several parameters. Measurements can be performed on the current image, the whole 3D stack if it is a 3D ROI, or across all frames. A result table (**Supplementary Fig. 12e**) is created with columns listing mean,

standard deviation, maximum, minimum and median values, as well as area/volume, centroid and center of mass.

- *Operations.* ROIs can be combined and logical operations can be applied (**Supplementary Fig. 12d**).
- *Draw.* ROIs can be freely placed and 'burned' onto any existing channel. On top new channels can be added for further ROI annotations. The user can choose between different colors for ROI borders and foreground (**Supplementary Fig. 12c**).

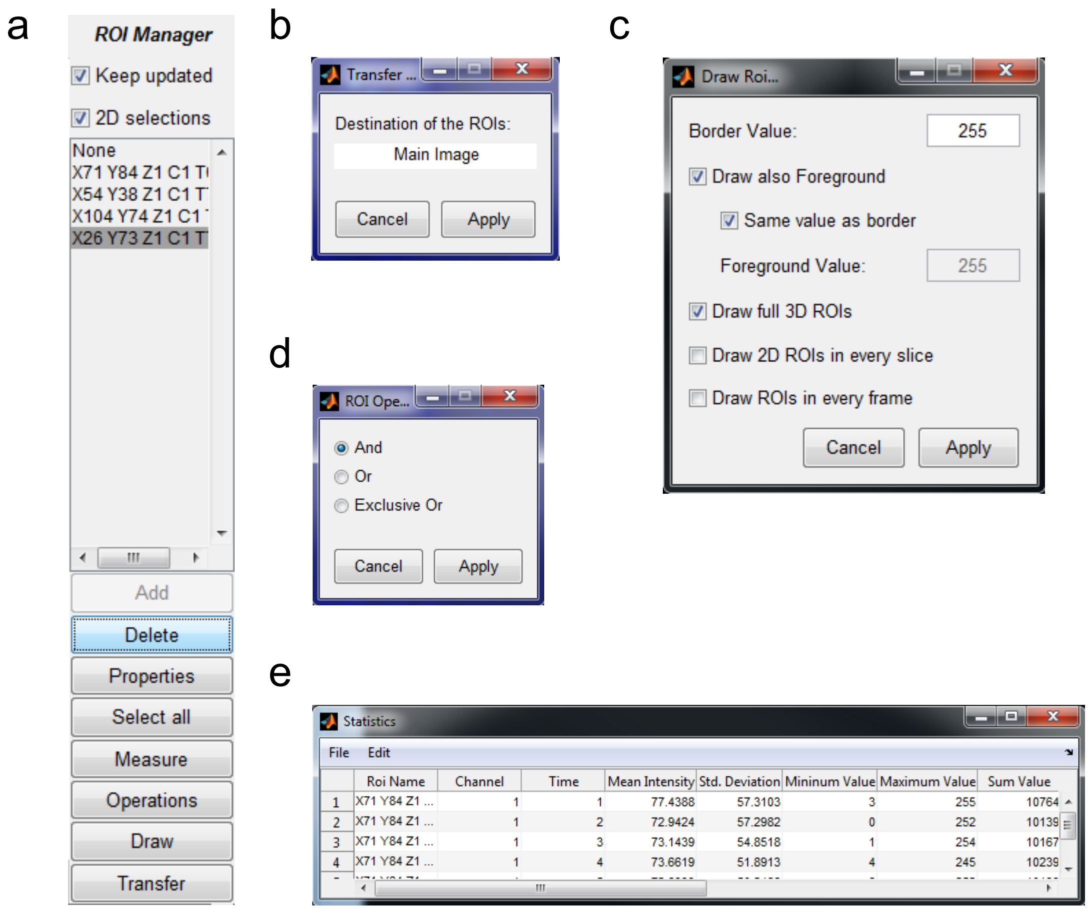

Supplementary Figure 12: ROI Manager related dialogs
a) ROI manager panel; b) ROI transfer dialog; c) ROI draw dialog; d) ROI operations dialog; e) Output measurements table

Open/Save Images

Only tagged image file format (TIFF) data can be read by the software. For non-TIFF files, conversion to TIFF is required. 2D maps and all other images are saved as BigTIFFs. Multiple channels can be saved in a single file or individually. In the first case, the largest bit-depth among the channels will be used.

Quantitative Analysis

Map3-2D is suitable for quantitative analysis, because it cross-references (both ways) between the data from the 2D map and the original image stack (**Figure 3 and Supplementary Fig. 5 & 6**).

As every map undergoes many mathematical operations like rounding, averaging and interpolation, the values on the map images are limited to extract precise quantitative information. However, Map3-2D completely solves this problem by allowing the user to freely select ROIs on the 2D map, transfer them to the original image stack and perform the measurements on the original data.

Objects with uneven surfaces: protrusions and indentations

In biology, it is generally not possible to find a reference ellipsoid that perfectly fits the sample under analysis. In many cases, the difference between the sample and the ellipsoid can be neglected and a standard map (like **Supplementary Fig. 4**) is sufficient for the evaluation. However, some objects exhibit protrusions and/or indentations from the reference ellipsoid, as shown in **Figure 2d**. For situations like this, the Map3-2D software provides an additional height channel (32 bits floating-point) at the output map, which can be considered as a map of elevations and valleys from the reference ellipsoid. In principle, this information serves as an indicator to evaluate the ellipsoid fitting. However, this tool can also be used to observe dynamic protrusions or indentations, and create height maps. Simulations of a moving protrusion and dynamic protrusion/indentation height changes are shown in **Supplementary Videos 1 and 2.** The videos show on top the rendering of the object together with the reference ellipsoid. Height maps onto Mollweide map projections are displayed at the center. Since height maps can be transferred in Map3-2D as an additional channel to the original stack, it can also be displayed as a volume-

rendered height channel, as shown at the bottom of the videos. An application to study nuclei shape dynamics is shown in **Supplementary Video 3**.

Supplementary References

1 Wolf, W. *et al.* Yeast Ist2 recruits the endoplasmic reticulum to the plasma membrane and creates a ribosome-free membrane microcompartment. *PLOS ONE* **7**, e39703 (2012).

2 Nyrtsov, M. V., Bugaevky, L. V. & Stooke, P. J. XXIII International Cartographic Conference (2007), available online at http://icaci.org/files/documents/ICC_proceedings/ICC2007/documents/doc/THEME%202/oral%201/2.1.5%20THE%20MULTIPLE%20AXIS%20ELLIPSOIDS%20AS%20REFERENCE%20S.doc.

3 Nyrtsov, M. V., Bugaevsky, L. M. & Shingareva, K. B. Mathematical basis for non-spherical celestial bodies maps. *J. Geospatial Engineering* **2**, 45-50 (2000).

4 Nyrtsov, M. V. & Stooke, P. J. The mapping of irregularly-shaped bodies at planetary scale. InterCarto8:GIS for sustainable development of territories. Proceedings from International Conference, Helsinki, 433-436 (2002).

5 Snyder, J. P. in Map projections: a working manual 4th edn (U.S. Government Printing Office, 1997).

6 Snyder, J. P. & Voxland, P. M. in An album of map projections (U.S. Government Printing Office, 1989).
